# An Exploration of Online Exercise Participation of Older Manitobans During the COVID-19 Pandemic

**DOI:** 10.1093/geroni/igaa057.3524

**Published:** 2020-12-16

**Authors:** Michelle Porter, Mikyung Lee, Ruth Barclay, Stephen Cornish, Nicole Dunn, Jacquie Ripat, Kathryn Sibley, Sandra Webber

**Affiliations:** University of Manitoba, Winnipeg, Manitoba, Canada

## Abstract

During the COVID-19 pandemic, in-person exercise programs for older people were temporarily closed, and some were replaced with online exercise. We explored the online exercise experiences of older people in Manitoba, Canada, using an online survey. We recruited a convenience sample (≥ 65 years), primarily through community organizations, and 745 people (57.5% female) consented. About 38.2% reported participating in online exercise during the pandemic. Most used pre-recorded classes (80.4%), from their local community (79.7%), and YouTube was the most used platform (57.4%). Almost all (82.7%) found the classes had the right variety and intensity. Of those who had participated in online exercise, 67.0% said they would participate in an online exercise class outside of a pandemic time. Participants like the following aspects better about online exercise: no transportation arrangements, it doesn’t matter what they wear, no travel time, and they like to exercise without others seeing them. However, they also miss being with and socializing with others, and they reported feeling unsafe when the instructor cannot see them. Of those who did not participate online, several reasons were given: they prefer to exercise with others in the same room, they prefer to exercise with an instructor directly present, no appropriate device, and their internet is not reliable. Many also provided examples of future circumstances when they might participate online, including: when the weather is not conducive to outdoor exercise, and if they can overcome technical issues. Lessons learned from this study can help those delivering online exercise in the future.

